# External Validation of the Derived Neutrophil to Lymphocyte Ratio as a Prognostic Marker on a Large Cohort of Pancreatic Cancer Patients

**DOI:** 10.1371/journal.pone.0078225

**Published:** 2013-11-04

**Authors:** Joanna Szkandera, Michael Stotz, Florian Eisner, Gudrun Absenger, Tatjana Stojakovic, Hellmut Samonigg, Peter Kornprat, Renate Schaberl-Moser, Wael AlZoughbi, Anna Lena Ress, Friederike Sophia Seggewies, Armin Gerger, Gerald Hoefler, Martin Pichler

**Affiliations:** 1 Division of Clinical Oncology, Department of Medicine, Medical University of Graz, Graz, Austria; 2 Clinical Institute of Medical and Chemical Laboratory Diagnostics, Medical University of Graz, Graz, Austria; 3 Division of General Surgery, Department of Surgery, Medical University of Graz, Graz, Austria; 4 Institute of Pathology, Medical University of Graz, Graz, Austria; Centro Nacional de Investigaciones Oncológicas (CNIO), Spain

## Abstract

**Background:**

With growing evidence on the role of inflammation in cancer biology, the presence of a systemic inflammatory response has been postulated as having prognostic significance in a wide range of cancer types. The derived neutrophil to lymphocyte ratio (dNLR), which represents an easily determinable potential prognostic marker in daily practise and clinical trials, has never been externally validated in pancreatic cancer (PC) patients.

**Methods:**

Data from 474 consecutive PC patients, treated between 2004 and 2012 at a single centre, were evaluated retrospectively. Cancer-specific survival (CSS) was assessed using the Kaplan-Meier method. To evaluate the prognostic relevance of dNLR, univariate and multivariate Cox regression models were applied.

**Results:**

We calculated by ROC analysis a cut-off value of 2.3 for the dNLR to be ideal to discriminate between patients’ survival in the whole cohort. Kaplan-Meier curve reveals a dNLR≥2.3 as a factor for decreased CSS in PC patients (p<0.001, log-rank test). An independent significant association between high dNLR≥2.3 and poor clinical outcome in multivariate analysis (HR = 1.24, CI95% = 1.01–1.51, *p* = 0.041) was identified.

**Conclusion:**

In the present study we confirmed elevated pre-treatment dNLR as an independent prognostic factor for clinical outcome in PC patients. Our data encourage independent replication in other series and settings of this easily available parameter as well as stratified analysis according to tumor resectability.

## Introduction

Pancreatic cancer (PC) is the ninth most common cancer but ranks forth as a cause of cancer-related mortality worldwide [1], [2]. Conventional chemotherapeutic agents have only modest effects on the course of the disease, and most patients survive less than one year after diagnosis [3], [4]. Currently, surgical resection represents the only curative option. Nevertheless, the prognosis of patients who undergo resection of PC with curative intention is generally poor unless they have early-stage disease, due to the fact that more than 85% of tumors have already extended beyond the organ margins at the time of diagnosis and invade the perineural spaces within and beyond the pancreas [5], [6]. Therefore, it is crucial to elucidate the biological mechanisms that contribute to tumor progression and identify prognostic factors that are helpful for patient’s counselling and individual risk assessment. Traditional prognostic factors, such as tumor size, histologic grade, vascular invasion, lymph node metastases, distant metastases and perineural invasion have been routinely used to predict outcome for PC patients [7]–[10]. These parameters are generally useful, however they are often insufficient in optimally predicting the individual patients’ prognosis. In the last years, many efforts were made to characterize novel immunological and histological prognostic markers. Although several potential molecular and cellular prognostic biomarkers have been described, their widespread routine application has not been established yet as most of them fail a successfully independent external validation. Nevertheless, external validation of prognostic risk assessment tools in independent cohorts of patients is paramount prior to the generalization of the applicability of a prognostic marker or model [11].

Tumor inflammation and immunology have recently been identified as enabling cancer characteristics, and increasing evidence supports their involvement in cancer progression and metastases [12]. The ability of the tumor to invade and metastasise depends on intrinsic characteristics of the tumor cells and the environment around the tumor [13]. Leucocytes, including neutrophils as well as lymphocytes, were reported to play an important role in tumor inflammation and immunology [13], [14]. There are several lines of evidence suggesting that an elevated peripheral blood neutrophil to lymphocyte ratio (NLR) is related to an adverse outcome in various types of cancer, including colorectal cancer (CRC), renal cell carcinoma, soft tissue sarcoma, non small cell lung cancer (NSCLC) and PC [15]–[20]. In clinical trials, solely the patients’ white cell and neutrophil counts are commonly entered into clinical trial databases. Therefore, Proctor and colleagues recently implemented a derived neutrophil to lymphocyte ratio (dNLR), which is composed of neutrophil count to (white cell count-neutrophil count). They evaluated the prognostic value of the dNLR on cancer outcome in different cancer types, and demonstrated that the dNLR had similar prognostic value to the well-established NLR [21]. However, they include a rather heterogeneous population of 700 patients with hepatopancreaticobiliary cancers and external validation of this prognostic risk assessment tool in independent cohorts has not been performed. Therefore, to validate the independent prognostic relevance of this cheap and easily determinable parameter, the present study was conducted to investigate the prognostic value of the pre-treatment dNLR on cancer specific survival (CSS) in a large cohort of patients with PC.

## Materials and Methods

This retrospective study included data from 474 consecutive patients with histological confirmed pancreatic adenocarcinoma, who were treated at the Division of Clinical Oncology, Medical University of Graz between 2004 and 2012. Patients where PC diagnosis was made by cytology or assumed by radiological assessment without proven histology from biopsy or surgical resection samples were not included in this study. Also other rare histological subtypes such as azinus cell carcinoma or neuroendocrine carcinoma were not included as they are associated with different prognosis [22]. All clinico-pathological data were retrieved from medical records at the Division of Clinical Oncology, as well as from pathology records from the Institute of Pathology at the same institution. Since the TNM classification system for PC changed during the study period, tumour stages were uniformly adjusted according to the 7th edition of this system [23]. Other documented clinico-pathological parameters included administration of chemotherapy with gemcitabine, gender and age. The laboratory data, including neutrophil, leucocyte counts and levels of tumour markers CA19-9 were obtained by exploration one week before treatment or histological proven diagnosis. Follow-up evaluations were performed every three months within the first three years, six months for five years and annually thereafter for curative resected tumor stages. For deceased patients, dates of death were obtained from the central registry of the Austrian Bureau of Statistics. The study was approved by the local ethical committee of the Medical University of Graz (No. 25–458 ex 12/13). As this is a retrospective non-intervention study, the institutional review board waived the need for written informed consent from the participants.

### Statistical Analyses

Cancer-specific survival was defined as the time (in months) from date of surgery or date of histological proven diagnosis to cancer-related death. First, the pre-published cut off value of 2 was applied for the continuous dNLR. Nevertheless, we seek an ideal cut-off value for the continuous dNLR by applying receiver operating curve analysis (ROC) analysis as previously reported [24]. The relationship between dNLR and other clinico-pathological parameters was studied by non-parametric tests. The patients’ clinical endpoints were calculated using the Kaplan-Meier method and compared by the log rank test. Backward stepwise multivariate Cox proportion analysis was performed to determine the influence of different clinico-pathological parameters on CSS. Hazard ratios (HRs) estimated from the Cox analysis were reported as relative risks with corresponding 95% confidence intervals (CIs). All statistical analyses were performed using the Statistical Package for Social Sciences version 20.0 (SPSS Inc., Chicago, IL, USA). A two-sided *p<0.05* was considered statistically significant.

## Results

Of the 474 patients with PC, there were 256 (54%) male and 218 (46%) female patients diagnosed with PC. The mean age at diagnosis was 64.5±10.4 years. Median survival was 7 months (range 0–79 months) and 408 (85.7%) patients died by their most recent follow-up visit. The mean dNLR was 2.77±4.19 and the median dNLR was 2.17. The AJCC tumor stage was defined as stage I in 5 patients, stage IIa in 18 patients, stage IIb in 85 patients, stage III in 33 patients and stage IV in 333 patients. Three hundred forty four patients underwent chemotherapy with gemcitabine. The patients who did not receive chemotherapy were either in a poor Karnofsky index, had contraindication with regard to co-morbidities or reject the recommended chemotherapeutic treatment. In 135 (28.5%) patients, a surgical resection of the tumor has been performed (see also Table 1).

**Table 1:** Clinico-pathological parameters of patients with ductal adenocarcinoma of the pancreas (n = 474).

Parameter	No. pancreatic cancer (%)
**Age at diagnosis (yrs.)**	
<65	220 (46.4)
≥65	254 (53.6)
**Gender**	
Male	256 (54)
Female	218 (46)
**Tumor stage**	
Stage I	5 (1.1)
Stage IIa	18 (3.8)
Stage IIb	85 (17.9)
Stage III	33 (7)
Stage IV	333 (70.3)
**Chemotherapy with gemcitabine**	
No chemotherapy	129 (27.2)
Adjuvant chemotherapy	75 (15.8)
Palliative chemotherapy	268 (56.5)
**dNLR**	
<2.3	203 (42.8)
≥2.3	271 (57.2)
**Tumor grading**	
G1+G2	291 (61.4)
G3+G4	183 (38.6)
**Surgical resection**	
No	339 (71.5)
Yes	135 (28.5)

dNLR, derived neutrophil to lymphocyte ratio.

Primarily, we validated the pre-published value of 2 as the cut-off for the continuous dNLR. To investigate whether a high dNLR was associated with clinical outcome in PC patients, univariate and multivariate analyses were performed. Univariate analysis identified older age (<65 versus ≥65, p = 0.009), high tumor stage (Stage I+II versus stage III versus stage IV, p<0.001), high tumor grade (G1 and G2 versus G3 and G4, p = 0.013), no chemotherapeutic treatment (no treatment versus chemotherapy, p<0.001), low Karnofsky index (<80 versus ≥80, p = 0.025), surgical resection (p<0.001) and increased dNLR ratio (<2 versus ≥2, HR = 1.37, CI95% = 1.12–1.67, p = 0.02) as prognosticators of poor outcome for patients’ CSS, whereas gender and level of CA19-9 were not statistically significantly associated with CSS (Table 2).

**Table 2:** Univariate and multivariate Cox proportional analysis regarding cancer-specific survival.

Parameter	Univariate analysis		Multivariate analysis	
	HR (95% Cl)	*p*-value	HR (95% Cl)	*p*-value
**Age at operation (yrs.)**				
<65	1 (referent)		1 (referent)	
≥65	1.30 (1.07–1.59)	0.009	1.05 (0.86–1.30)	0.626
**Gender**				
Male	1 (referent)		1 (referent)	
Female	1.13 (0.93–1.38)	0.218	1.06 (0.87–1.31)	0.556
**Tumor stage**				
Stage I+II	1 (referent)		1 (referent)	
Stage III	3.06 (1.88–4.97)	<0.001	2.18 (1.23–3.87)	0.008
Stage IV	3.89 (2.94–5.15)	<0.001	3.37 (2.02–5.61)	<0.001
**Tumor grade**				
G1+G2	1 (referent)		1 (referent)	
G3+G4	1.29 (1.06–1.58)	0.013	1.72 (1.39–2.12)	<0.001
**Chemotherapy**				
No	1 (referent)		1 (referent)	
Yes	0.42 (0.34–0.52)	<0.001	0.34 (0.27–0.43)	<0.001
**CA 19-9**				
<median level	1 (referent)		1 (referent)	
≥median level	1.02 (0.99–1.05)	0.237	1.01 (0.98–1.04)	0.532
**dNLR**				
<2.3	1 (referent)		1 (referent)	
≥2.3	1.53 (1.25–1.86)	<0.001	1.24 (1.01–1.51)	0.041
**Surgical resection**				
No	1 (referent)		1 (referent)	
Yes	0.33 (0.26–0.43)	<0.001	0.77 (0.49–1.21)	0.256
**Karnofsky Index**				
<80	1 (referent)		1 (referent)	
≥80	0.94 (0.88–0.99)	0.025	0.96 (0.90–1.03)	0.218

dNLR, derived neutrophil to lymphocyte ratio.

**Figure 1:**
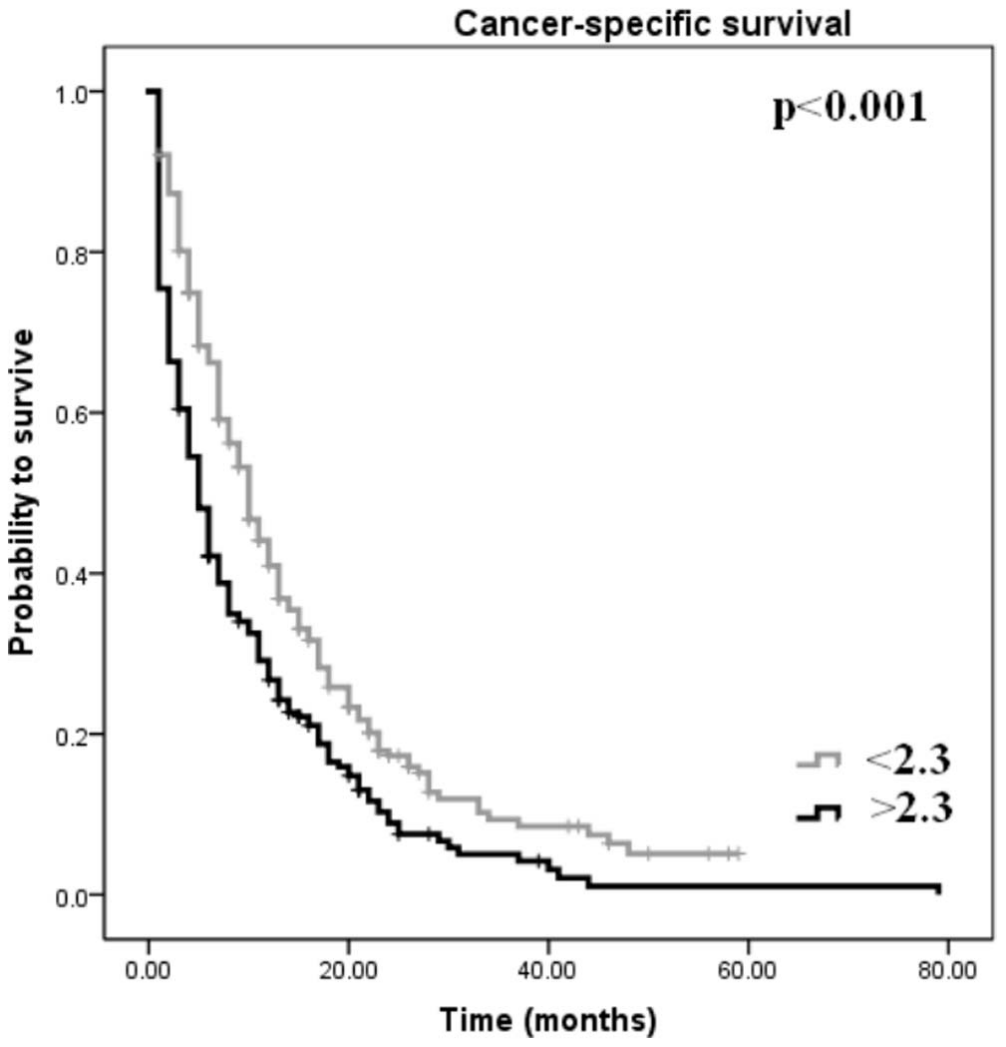
Kaplan-Meier curve for cancer-specific survival regarding high (≥2.3) versus low (<2.3) derived neutrophil to lymphocyte ratio (p<0.001).

Furthermore, high dNLR was significantly correlated with high tumor stage, lower rate of surgical resection, higher CA19-9 levels and the lack of administration of chemotherapy (p<0.05), whereas no association with gender, age and tumor grade could be found (data not shown). To determine the independent prognostic value of the dNLR for CSS, a multivariate analysis using a Cox proportional hazard model was performed. In the multivariate analysis that included age, gender, CA19-9 levels, tumor stage, tumor grade, administration of chemotherapy, Karnofsky index, surgical resection and dNLR, we identified tumor stage (p<0.001), tumor grade (p<0.001) and administration of chemotherapy (p<0.001) as independent prognostic factors for CSS, whereas the other parameters including the dNLR ratio (cut off at 2) were not significantly associated with CCS (HR = 1.05, Cl95% = 0.85–1.29, p = 0.64). Therefore, applying the criteria mentioned above, we determined by using ROC analysis a cut-off value of 2.3 for the dNLR to be best to discriminate between patients’ survival in the whole cohort. This cut-off value prompted us to re-evaluate the dNLR as a universally useful prognostic biomarker in our study cohort. Figure 1 shows the Kaplan-Meier curves for CSS and reveals that a dNLR≥2.3 is a consistent factor for decreased CCS in PC patients (p<0.001, log-rank test). To determine the independent prognostic significance of the new established cut off value of dNLR for CSS, a multivariate Cox proportional hazard model including age, gender, CA19-9 levels, tumor stage, tumour grade, administration of chemotherapy, Karnofsky index, surgical resection and dNLR was calculated. In the multivariate analysis, we identified tumor stage (p<0.001), tumor grade (p<0.001), administration of chemotherapy (p<0.001) and the dNLR (<2.3 vs. ≥2.3;, p = 0.041) as independent prognostic factors for CSS (Table 2).

## Discussion

In the present study, we externally validated for the first time the dNLR in patients with PC and found a significant association between an elevated pre-treatment dNLR and poor clinical outcome. A high dNLR reflects an increased neutrophil and/or a decreased leucocyte ratio. It is generally accepted that inflammatory processes in the tumor microenvironment play a crucial role in promoting proliferation, invasion and metastasis of malignant cells [13], [14]. The infiltrating leucocytes, including neutrophils and lymphocytes, are important factors in this process [13]. Neutrophilia has been associated with malignancy. However, the cause is not completely understood. Neutrophils in peripheral blood or in the tumor microenvironment were shown to produce pro-angiogenic factors including vascular endothelial growth factor to stimulate tumor development and progression [25]. The cytokines involved in cancer-related inflammation, including interleukin-6 (IL-6) and tumor necrosis factor-alpha (TNFα), may induce neutrophilia [26], [27]. The para-neoplastic production of myeloid growth factors by cancer cells may represent an additional cause of neutrophilia [28]. Hence, a high peripheral neutrophil level may indicate a cancer-related inflammation or tumor progression, and predict poor clinical outcome. Besides the neutrophils, leucocytes are mostly composed of lymphocytes. Immune cells that infiltrate into or around the tumor engage in dynamic and extensive crosstalk with cancer cells [29]. Over the past decade, there has been growing evidence that lymphocytes operate as crucial components of the adaptive immune system and are the cellular basis of cancer immunosurveillance and immunoediting [30]. Furthermore, infiltrating lymphocytes have been reported to indicate the generation of an effective anti-tumor cellular immune response [31]. Therefore, a low lymphocyte count may be responsible for an inadequate immunologic reaction to the tumor, and consequently a weakened defence against cancer, resulting in poor prognosis [32]. Activated specific CD8+ T cells were shown to control tumor growth by cytotoxic activity and inducing apoptosis of tumor cells [33]. CD4+ T cells are crucial for screening cytokines such as IL-2, which are essential for CD8+ T cell growth and proliferation. Furthermore, recent reports reveal that activation of CD4+ T cells is required for immunization of CD8+ T cells against cancer [34]. In vitro studies showed that the cytolytic activity of lymphocytes and natural killer cells was suppressed when co-cultured with neutrophils, and the extent of suppression was proportionally enhanced to the addition of neutrophils [35], [36]. Accordingly, an elevated pre-treatment NLR was reported to correlate with reduced survival in several types of cancer. For instance, in 177 PC patients, an elevated NLR was superior to other inflammation-based prognostic scores in the prediction of reduced OS [19]. These findings were in line with a smaller study including 74 PC patients, showing a decreased disease-free survival in patients with a high NLR [20]. Taken together, these results demonstrate that the NLR may serve as a prognostic factor in different types of cancer. However, there is plenty of clinical trial data, where only white cell and neutrophil counts have been recorded in computer databases. Therefore, Proctor et al evaluated the prognostic value of the dNLR in a large cohort of 12.118 patients with different cancer types, including pancreatic cancer, and clearly demonstrated that the dNLR has a similar effect on prognosis as the NLR, showing a poor clinical outcome in patients with elevated dNLR, and that it can be equally used to predict survival [21]. In our study, we first externally validated the pre-published cut-off value of 2, determined for the dNLR in the study by Proctor et al., and found a statistically significant association between dNLR≥2 and decreased CSS in univariate, but not in multivariate analysis. Procter and colleagues determined their optimal cut off value by calculating the whole set of several different cancer types. Therefore, we determined a cut-off value of 2.3 for the dNLR to be optimal for the specific cohort of PC patients. We found a statistically significant association between dNLR≥2.3 and poor clinical outcome in multivariate analysis, highlighting the independent value of this parameter. These results indicate that the dNLR has considerable potential as a prognostic marker for the examination of risk stratification of patients in all current clinical trials in PC. Furthermore, if confirmed in other independent series, it can also be used as an easily available and inexpensive marker in daily clinical practice to guide individualized treatment decisions in patients with PC. Also importantly, tumor stage, tumor grade and being able to receive chemotherapy remain the most significant prognostic data for cancer specific survival at the multivariate analysis, indicating to the high quality of our database. In this context, dNLR adds some prognostic information to the well-established factors, but will neither perform better nor represent a substitute for one of them. In our study, some limitations have to be taken into account as this is a retrospective data collection with no prospective study design. There is also a high risk of over fitting of data (multivariable Cox analysis was performed after ROC curved). The study also needs to be replicated in independent series. However, for the first time, we externally validated the prognostic value of the dNLR at the proposed cut off level of 2 and could not confirm the results of Proctor and colleagues in a large cohort of PC patients. According to our data, in ductal adenocarcinoma of the pancreas, a cut off of 2.3 seems to be optimal and should be further validated in a prospective manner.

In conclusion, our study provides evidence that pre-treatment dNLR can be considered as a promising independent prognostic parameter in PC patients. Further independent prospective trials are warranted to confirm these results.
